# Supplementary material for: Enhanced voice recognition in musicians
Source: PLoS One. 2025 May 19;20(5):e0323604. doi: 10.1371/journal.pone.0323604 (PMC12088071; doi:10.1371/journal.pone.0323604)
Supplement: Attachment — Submitted filename: Response to Reviewers.pdf [file pone.0323604.s002.pdf]

Enhanced voice recognition in musicians

Allison J. Sletcher, Stefania S. Moro, and Jennifer K. E. Steeves*

Response to Reviewers

Journal Requirements:

1. When submitting your revision, we need you to address these additional requirements.

Please ensure that your manuscript meets PLOS ONE's style requirements, including those for file naming. The PLOS ONE style templates can be found at https://journals.plos.org/plosone/s/file?id=wjVg/PLOSOne_formatting_sample_main_body.pdf and https://journals.plos.org/plosone/s/file?id=ba62/PLOSOne_formatting_sample_title_authors_affiliations.pdf

All requirements have been addressed.

2. Thank you for stating the following financial disclosure: "Canada First Research Excellence Fund: Vision Science to Application (VISTA) (CFREF-2015-00013); Natural Sciences and Engineering Research Council of Canada (NSERC) (327588); Canada Foundation for Innovation (CFI) (12807). "

Please state what role the funders took in the study. If the funders had no role, please state: "The funders had no role in study design, data collection and analysis, decision to publish, or preparation of the manuscript."

If this statement is not correct you must amend it as needed.

Please include this amended Role of Funder statement in your cover letter; we will change the online submission form on your behalf.

The statement is correct and has been added to the cover letter to be updated in the online submission system.

3. Thank you for stating the following in the Acknowledgments Section of your manuscript: "We sincerely thank all participants for taking part in this study. This research was supported by grants from Canada First Research Excellence Fund: Vision Science to Application (VISTA) (CFREF-2015-00013); Natural Sciences and Engineering Research Council of Canada (NSERC) (327588); Canada Foundation for Innovation (CFI) (12807) to JKES."

We note that you have provided funding information that is not currently declared in your Funding Statement. However, funding information should not appear in the Acknowledgments section or other areas of your manuscript. We will only publish funding information present in the Funding Statement section of the online submission form.

Please remove any funding-related text from the manuscript and let us know how you would like to update your Funding Statement. Currently, your Funding Statement reads as follows: "Canada First Research Excellence Fund: Vision Science to Application (VISTA)

(CFREF-2015-00013); Natural Sciences and Engineering Research Council of Canada (NSERC) (327588); Canada Foundation for Innovation (CFI) (12807)."
Please include your amended statements within your cover letter; we will change the online submission form on your behalf.
Funding information has been removed from our manuscript acknowledgment section. The funding information provided in the funding statement in the online submission form is accurate.

4. We note that Figure 1 includes an image of a participant in the study.

As per the PLOS ONE policy (http://journals.plos.org/plosone/s/submission-guidelines#loc-human-subjects-research) on papers that include identifying, or potentially identifying, information, the individual(s) or parent(s)/guardian(s) must be informed of the terms of the PLOS open-access (CC-BY) license and provide specific permission for publication of these details under the terms of this license. Please download the Consent Form for Publication in a PLOS Journal (http://journals.plos.org/plosone/s/file?id=8ce6/plos-consent-form-english.pdf). The signed consent form should not be submitted with the manuscript, but should be securely filed in the individual's case notes. Please amend the methods section and ethics statement of the manuscript to explicitly state that the patient/participant has provided consent for publication: “The individual in this manuscript has given written informed consent (as outlined in PLOS consent form) to publish these case details”.
If you are unable to obtain consent from the subject of the photograph, you will need to remove the figure and any other textual identifying information or case descriptions for this individual.
The face image in figure 1 is not a participant. This is an image of a stimulus that was used during the study. All faces used as stimuli have been modified from their original form. Models gave written informed consent to use their images in our study and for subsequent use in publications and presentations. We have included the relevant text above in our manuscript both in text and in the caption for figure 1.

Reviewer #1: Overview

The goal of this paper is to evaluate whether the neuroplastic benefits of music training on auditory skills extend to object-sound and face-voice recognition following brief identification training. In general, I think this study has the potential to provide nuance to the effects of music training on perceptual abilities. However, I have significant concerns about the statistical analyses that potentially call into question the validity of the results and may limit overall conclusions.

Major comments

Introduction

1. It seems that "voice processing" is used interchangeably with notions of speech processing in the introduction and discussion, but these ideas are quite distinct. While the included citations are used to support theories of musical training effects on the brain, it creates confusion about the motivation for the study and in some cases appears to misinterpret findings across the literature. For example:
a. (lines 63-66) "A relationship between musical training and voice processing at a cortical level has also been demonstrated, where enhanced and earlier cortical responses to syllabic duration and voice onset time has also been measured in children." But syllabic duration and voice onset refer to processing of speech phonemes, not properties of voices, per se
b. (lines 261-263) "For example, faster learning of voices in unfamiliar languages and enhanced cortical and subcortical responses to auditory stimuli has also been correlated to years of musical training in musicians." – but several of these citations are about processing speech phonemes, not voice recognition

To aid clarity, I suggest reframing those citations as "speech processing" and include more relevant literature for "voice processing" as it pertains to voice recognition and learning to distinguish timbre or other voice characteristics which would be more pertinent for the current study.
Thank you for your suggestions. We have reframed the citations mentioned in points A and B as "speech processing" and have expanded our discussion of voice processing in our introduction. All modifications to the introduction and discussion have been made using track changes. A copy of the added discussion on voice processing has also been copied below:

These cues are common to both linguistic and voice processing and could provide a strong foundation for musicians to develop superior voice recognition. Speech and language tasks, such as speech-in noise recognition (ie. "cocktail party" scenarios) may be improved by musical training, by strengthening shared resources[for review see 28] *through increasing the listening capacity in both ideal acoustic conditions and also difficult acoustic environments.*[for review see 29] *Training studies on speech-in noise perception*[28] *and experience dependent plasticity in the auditory system*[30 - 32] *suggest that musical training*

can provide long lasting benefits to auditory function including simple perceptual enhancements and factors impacting higher order cognition such as working memory and intelligence.[for review see 33, 34] However, genetic[35] and epigenetic factors (such as behavioural traits like personality,[36] motivation,[37] and the interaction between factors[38] are also likely to contribute to musical and speech-in noise cognition.[35]

2. Additionally, the introduction does not go far enough in motivating the current research question, in my opinion. One area that would benefit from more details is literature on human voice processing vs. non-human auditory objects. The only sentence I can find in the intro is "Musicians demonstrate better auditory memory for spoken words and environmental sounds,35, for review see 36 but they do not demonstrate better visual memory for object categories.35, 37" (lines 71-73). The introduction up to this point focused mainly on voice recognition (though see previous comment), so it comes a bit as a surprise to me when object recognition is suddenly mentioned in the last paragraph. Additionally, the enhanced skills in "pitch, timbre, and tempo of music" due to music training might also theoretically extend to object recognition. Even though the findings reportedly suggest otherwise, more details in the intro would plant a seed for possible similarities, differences, and musical training effects on processing voices vs. non-voice stimuli.
Thank you for your insightful comment. We have expanded the introduction to include a discussion of human voice processing compared to non-human auditory objects. All modifications have been made using track changes. A copy of the added discussion on voice vs object sound processing has also been copied below:

The specialization of auditory processing in musicians may also extend to sound recognition more broadly. Musicians demonstrate better auditory memory for spoken words and environmental sounds,[54, for review see 56] but they do not demonstrate better visual memory for object categories.[35, 37] Cohen and colleagues (2011) found musicians had better auditory memory for spoken words and environmental sounds compared to non-musicians for an object category recognition task. Typically, in object recognition studies, objects are presented from a range of object categories followed by a memory recognition task to investigate the perceptual processing of general object recognition as opposed to recognition of a specific object representation.[54, 56] To date, there have been no studies investigating whether musicians have enhanced auditory sensitivity for specific object identity recognition. The current study asks whether musical expertise contributes to person and object recognition when both auditory and visual cues to identity are available.

3. The citations for neural processes & musicianship do not fully reflect the current state of the literature. I recognize the current study is not a neuroimaging study, and the generic statements about neural processes and music training-induced neuroplasticity are merely to support a case for possible transfer effects to voice & object recognition. However, I think the current manuscript is missing (or perhaps misrepresenting) important considerations/nuance from the body of work that was already briefly cited.
a. For example, the intro cites some studies in relation to subcortical processes (mainly FFR; lines 58-62), but more recent work has pointed out the cortical contributions with low

stimulus F0s (e.g., Bidelman 2018, NeuroImage)—relevant for the cited older Kraus work—or MEG methodology (e.g., Coffey et al 2016, Nature Communications). Cortical vs. subcortical processes may be under different learning timescales or constraints, whether in terms of the short-term experiences like the experimental design presented here or for longer-term experiences like musical training.
b. Additionally, some of the musicianship-FFR literature may need to be revisited in light of recent evidence that suggests myogenic responses (postauricular muscle artifact) might contribute to the musician-related differences observed in FFRs (Bidelman et al 2024, Frontiers in Neuroscience).
c. (lines 62-63) While years of music training relationships are more suggestive of a training effect rather than mere categorical group differences, it does not rule out inherent, genetic predispositions that could have contributed to differences in neural responses (e.g., Mankel & Bidelman, 2018, PNAS), speech processing behaviors (e.g., Swaminathan & Schellenberg 2017, Psychonomic Bulletin & Review), voice emotion recognition (Correia et al 2022 Emotion), the desire to pursue musical activities in the first place (e.g., Wesseldijk et al 2021, Psychological Science), and/or to stick with the training for longer than less-musically-inclined peers (see also review by Schellenberg & Lima 2024, Annual Review of Psychology).
i. For further discussions on genetic contributions, see: Wesseldijk et al 2023, Translational Psychiatry; Tan et al 2014, Frontiers in Psychology; Mosing et al 2014, Psychological Science; Hambrick & Tucker-Drob 2015, Psychonomic Bulletin & Review; Wesseldijk et al 2019, Developmental Psychology; etc.

Thank you for your suggestions. We have expanded our introduction to address each of your suggestions above. We have used track changes to highlight the new additions to the literature review. A copy of the added text has also been copied below:

Musical training appears to be associated with plasticity in both cortical and subcortical brain regions that process pitch, duration, and onset time of voice stimuli.[For review see 39] *For example, when presented with linguistic pitch patterns musicians have enhanced and more accurate frequency encoding in the inferior colliculus.*[40] *It has also been shown that musicians have modulated inter-regional neural communication compared to non-musicians.*[41, 42] *For example, in music/speech categorical processing musicians had increased activation in early primary auditory cortex whereas less experienced non-musicians had increased activation in downstream, higher-order linguistic brain areas such as the inferior frontal gyrus.*[41] *These findings indicate that cortical and subcortical processes may be under different learning timescales or constraints whether in terms of short-term experiences or for long-term experiences such as musical training. Recent evidence suggests that the enhanced frequency-following response (a scalp-recorded neuroelectric brain recording that serves as a neural index of sound encoding in EEG) may be impacted by myogenic responses, such as a postauricular muscle artifact which may contribute to the musician related differences observed in frequency-following responses.*[43]

Cortical and subcortical responses have been correlated to years of musical training[39, 40, 44] *establishing a relationship with length of musical training. A relationship between musical training and speech processing at a cortical level has also been*

demonstrated, where enhanced and earlier cortical responses to syllabic duration and voice onset time has also been measured in children.[45, 46] Finally, years of musical training is positively correlated to faster learning of voice identification in a non-native language.[47] English and Mandarin speaking musicians have an advantage for voice identity recognition for unfamiliar languages that may be attributed to superior pitch processing abilities.[48] While years of music training relationships are suggestive of a training effect, recent studies have demonstrated that inherent, genetic predispositions may also contribute to differences in neural responses,[49] speech processing behaviours,[50] voice emotion recognition,[51] the desire to pursue musical activities,[52] and to commit to training for longer than less musically inclined peers.[53 for review]

Methods

4. Certain aspects of the methods are missing or not well described, making it difficult to evaluate the rigor and potentially limiting reproducibility of the study:
a. Even if the stimuli were controlled for RMS amplitudes, background noise, distinguishing features, etc. as mentioned in the methods, characteristic differences across the stimuli could still artificially inflate identification performance. Were the objects, faces, and associated audio validated for equivalent identification performance? Did all the voices speak the same English phrase? Do the car horns all have unique timbres and pitch contours, or are the pitch contours similar?
For this task objects, faces, and associated auditory stimuli were not separately validated for equivalent identification performance. Individual variability was reduced across all stimuli, both learned and new, to control for external factors such as background noise and distinguishing features. All voices spoke the same English phrase. This has been clarified in the manuscript using track changes. Car horns had unique timbres and pitch contours. All stimuli have been previously used in other published studies (Hoover et al., 2010 and Moro et al., 2019). In addition, since the focus of this paper is a between groups design, each group was presented with the same stimuli and any potential variation between auditory characteristics across categories or conditions would be equal across groups.

i. The cited Hoover et al (2010) previously reported sensitivity differences across the voice and horn stimuli. Thus, it is possible that the stimuli are not equated in identification difficulty across conditions which could impact the group effects. For example, if there is already increased sensitivity to the horn sounds compared to voice recognition, perhaps there was not enough "room" or variation for music training to demonstrate a group-wise effect in this study, even if underlying perceptual advantages were present in this group.
ii. However, it does not seem the same effects are replicated here, at least not to the same degree. It would be pertinent to address these apparent deviations from previous studies using the same stimuli in the discussion.
There was no ceiling effect for either group for auditory sensitivity for either condition (see figures 3 and 4). We have conducted a comparison for each group (musicians and non-musicians) to investigate whether there is a difference between voice and horn recognition sensitivity across tasks (person and object identification). All data was normally

distributed, therefore parametric paired samples t-tests were conducted. We have added this comparison to our results and have included a new figure illustrating these results. We have also addressed this in the discussion of our paper using track changes. A copy of the updated results section also been copied below:

3.5 Comparison Across Voices and Horns

To determine whether there was an effect of the type of auditory stimulus (Voices or Horns) we conducted parametric paired samples t-tests to compare the auditory sensitivity scores of Musicians and Non-musicians across the person identity and object identity tasks. Musicians did not differ in their auditory sensitivity for voices (M = 2.38, SD = 0.54, 95% CI [2.20, 2.57]) compared to object sounds (M = 2.40, SD = 0.66, 95% CI [2.16, 2.63]), t(34) = -0.125, p = 0.90, d = -0.02. Non-musicians demonstrated a strong trend for enhanced auditory sensitivity for voices (M = 2.05, SD = 0.61, 95% CI [1.84, 2.26]) compared to object sounds (M = 2.34, SD = 0.69, 95% CI [2.10, 2.58]), t(34) = -2.031, p = 0.05, d = -0.34.

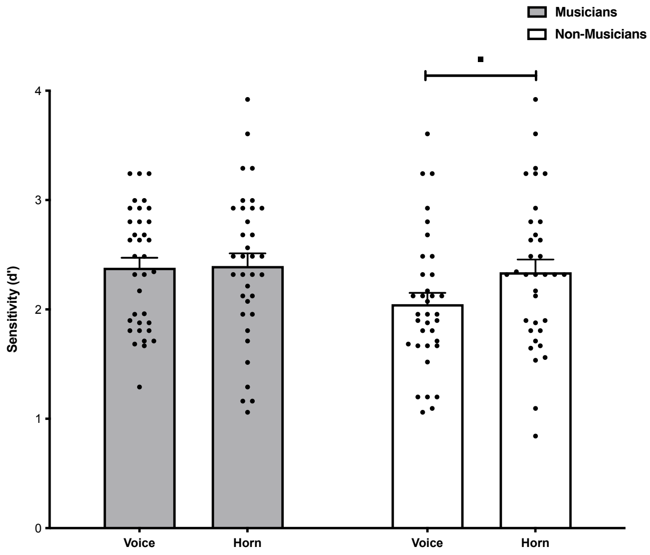

Figure 5. *Auditory only stimulus sensitivity in the person identification (face/voice) and object identity (car/horn) tasks for Musicians (grey bars) and Non-musicians (white bars). Error bars represent standard error of the mean.*

b. In the bimodal block, it is not clear whether the correct response for an incongruent pair is "new" or if at least one cue is learned then the correct response is "learned". Based on the previous work by Hoover et al, I think the latter qualifies as a "new" pair, but it would still be helpful if this was more clearly described in the current paper.
You are correct, an identity consisting of one modality that was learned paired with another modality that is new would qualify as a "new" pair. This has been clarified in the Procedure section using track changes.

c. The stats are missing critical details on data cleaning procedures, ANOVA assumptions (normality, homogeneity of variance), how influential outliers evaluated, etc. Failure to conduct a thorough evaluation of the data and rule out potential statistical assumption

violations could contribute to the seemingly discrepant non-significant interaction but significant group x auditory post hoc comparison for the person identity recognition block. A more detailed description of the data checking procedures would at least increase confidence in the results.
These details have been added to the relevant results sections using track changes. There were no violations of assumptions and parametric repeated measures ANOVAs were conducted. The hours of weekly practice and years of musical training variables violated normality and we updated our results to include non-parametric Spearman rank-order correlation analyses. The results of each correlation did not change from our original version. All modifications have been made in the results section using track changes.

d. Based on my understanding of d', the mean sensitivity scores >2 or >3 are good indicators or near-optimal/ceiling performance across the board. However, line 155 states "Participants with perfect scores were adjusted". Although a citation is listed at the end of this sentence, I think this is a case where a full description of the exact methodology is needed for readers to understand exactly how scores were adjusted to know its potential impact on the results. How many scores needed adjustment for each condition or in each group?
This information has been added to the manuscript. There were no scores of 0, therefore the adjustment (+/- 0.025) was only applied to reduce scores from ceiling. The text has been copied below:

Participants with perfect scores were adjusted based on recommendations by Macmillan and Creelman,[60] by replacing their perfect score with a constant (+/- 0.025). Approximately 10% of the data for the person identification task and 9% of the data for the object identification task was adjusted in this manner.

Results & discussion

5. It is debated whether post hoc tests should be conducted on an interaction term when the global group x condition interaction itself is not significant for person identity recognition. Since the global F-test and the post hoc t-tests technically assess different hypotheses, of course it is theoretically possible to get a significant post hoc without a significant F-test (Midway et al 2020, PeerJ). Yet, others argue that continuing to test post hocs on a non-significant interaction term can further inflate Type I error even if using post hoc correction factors (Nieuwenhuis et al 2011, Nature Neuroscience; Garofalo et al 2022, PLOS ONE). Further, the means of each group are within 1 SD of each other. I am thus highly skeptical of the Group effects in the Auditory-only condition for the voice identity recognition block. Conclusions drawn from this result seem tenuous at best and should therefore be interpreted more carefully.
We have modified our interpretations to be more careful and less generalized in our discussion. All edits have been made using track changes.

6. One suggested approach might be to include 95% confidence intervals in addition to (or

in place of) the post hoc analyses to reduce likelihood of Type I error (Garofalo et al 2022, PLOS ONE).
Confidence intervals have been added to the analyses in addition to the post hoc analyses. Results section has been updated using track changes.

7. Running several similar correlations on subsets of the data as in Table 1 without corrections for family-wise multiple comparisons are also at risk for Type I error (Curtin & Schulz 1998, Biological Psychiatry). From what I can tell, a simple Bonferroni correction would make most if not all of the starred correlations in Table 1 no longer significant.
We understand your point about the importance of correcting for multiple comparisons by controlling for family-wise error rate. We have revised our correlation analysis to decrease the number of variables that we are correlating that are not relevant to our hypotheses, namely sensitivity to the visual stimuli and sensitivity to the bimodal stimuli. By including these extra variables, we acknowledge that we would be contributing to increasing the type I error rate with variables that are not relevant to this analysis. We have also amended our results section to indicate that the p-values are not adjusted for clarity. The relevant text has also been copied below:

3.3 Relationship Between Musical Experience and Auditory Sensitivity

Spearman rank-order correlation analyses were conducted to examine the relationship between musical experience and auditory sensitivity for musicians and non-musicians. The musical experience variable (Years of Musical Training) was not normally distributed therefore a non-parametric analysis was conducted (Non-Musician: $W = 0.25$, $p < 0.001$; Musicians: $W = 0.94$, $p = 0.07$). There was a small positive correlation between years of training and performance sensitivity, where more training experience is related to higher sensitivity for voices in person recognition. Further, there was no relationship between years of training and object sound recognition across all stimulus condition modalities. See Table 1 for a breakdown of the correlation results.

3.4 Relationship Between Hours of Weekly Practice and Auditory Sensitivity

Spearman rank-order correlation analyses were conducted to examine the relationship between hours of weekly musical practice and auditory sensitivity for musicians and non-musicians. The hours of weekly musical practice variable was not normally distributed therefore a non-parametric correlation analysis was conducted (Non-Musician: $W< 0.00$, $p < 0.001$; Musicians: $W = 0.830$, $p < 0.001$). There is no relationship between hours of weekly practice and sensitivity to voices for person identification. There was no relationship between hours of practice and object sound recognition. See Table 1 for detailed Spearman correlation results.

Table 1. *Spearman rank-order correlations examining the relationship between years of training and hours of weekly practice for auditory person and object identity recognition sensitivity. Significant correlations are indicated with an asterisk (p-values are not adjusted for multiple comparisons).*

	Auditory Sensitivity	
	ρ	*p*
	Years of Training	
Person Identity Recognition	*0.26*	*0.03**
Object Identity Recognition	*0.05*	*0.67*
	Hours of Weekly Practice	
Person Identity Recognition	*0.30*	*0.01**
Object Identity Recognition	*0.07*	*0.57*

8. In light of the stats/results concerns listed above, I feel the effects of music training on voice/object recognition are not robust enough, or perhaps the experimental paradigm was not quite sensitive enough to these group differences in a manner that survives more stringent multiple comparisons. As a result, this study reads as rather exploratory, and I believe more caution should be expressed throughout the entire manuscript when interpreting the results.
We have revised our discussion to interpret our results with more caution.

Minor comments

9. (lines 50-53) "For example, linguists, speech pathologists, and musicians who spend more time evaluating auditory stimuli than the average person may become auditory specialists. Highly trained forensic voice experts have superior voice discrimination skills when asked to identify a target voice from a "lineup" of voices. Musicians have established skills…" I think the inclusion of the forensic voice experts sentence creates an awkward flow in this paragraph. The previous sentence is a setup about musicians with additional details about musicians in the sentence afterwards, but the continuity of thought is broken up by the forensic experts sentence. Suggest rephrasing.
This has been rephrased using track changes. The text has also been copied below:

Superior voice recognition may be associated with accentuated auditory skills and specialization of auditory cortical pathways through training. For example, highly trained forensic voice experts have superior voice discrimination skills when asked to identify a target voice from a "lineup" of voices.[20, 21] *Linguists, speech pathologists, and musicians who spend more time evaluating auditory stimuli than the average person may also become auditory specialists.*[19]

10. Is there a model number for the SONY headphones? It is possible that some consumer-grade headphone response properties filter out certain frequencies, perhaps with mildly degraded auditory quality that would otherwise aid discrimination of the auditory stimuli. It would be helpful for future replications of this work to be certain of the auditory equipment used.

The headphones used for the study were SONY noise cancelling headphones model #: MDR-ZX110NC. The model number has been included in the methods section using track changes.

11. The mean stimulus intensity level for the auditory sounds (or range) should be added to the methods.
The mean stimulus intensity level for the auditory sounds and range has been added to the methods using track changes.

12. “visual processing is superior” (line 178), “visual sensitivity is superior” (line 198) is odd phrasing, stating superiority of a modality. I recommend modifying these phrases to something like “performance/accuracy was higher overall for visual domain” or “higher sensitivity to the visual domain” or something similar to avoid the implication of modality “superiority”.
We have modified these conclusions to reflect your suggestion using track changes.

13. I think some of the concerns above about the data would be partly resolved if another plot type was used to show the spread of the data (e.g., a strip chart overlaid on the bar chart, violin plots, histograms next to the bar charts, etc.).
The figures have been updated to include strip charts overlaid on the bar chart.

Reviewer #2:

To Editor and Authors, this manuscript describes a behavioural experiment comparing Musician to Non-musician participants at an old/new recognition task with Person (face/voice) or Car (car/honk) stimuli presented in 3 versions: auditory, visual, or both modalities. Participants first learned an auditory-visual association task with both the Person or Car stimuli, then were tested with the 3 versions (auditory, visual, audiovisual) of the old/new task. Results indicate a sensitivity difference between musicians and non-musicians for recall of the auditory stimuli only, correlating mildly with years of musical training in Musicians. This result corroborates past results showing that Musicians have better timbre recognition in general. The article is well-written, with proper reference to the literature, and clean statistical analyses.

A major problem I see is that there is no clear relation to multimodality – the main purpose of the study. Indeed, the audiovisual task could be solved by participants either by detecting deviance from learned associations (true audio-visual association learning) OR by detecting novel stimuli in either modality without regard to cross-modal congruence. Indeed the task could be solved either by recognizing that the presented association was not one of the learned ones (true bimodal learning) OR by detecting novelty in either modality – a much easier task not relying on audio-visual integration. It is not clear form the data which of these two processes the participants engaged in. This complexity could have been avoided by using incongruent audiovisual pairings composed of old, although incongruent stimuli – not novel ones. This does not change much about the interesting result of superior voice recognition in musicians, but only seems as a side result given the initial multimodal scope of the project.
Thank you for your comments. We have modified our results and discussion sections to emphasize the findings regarding the unimodal auditory sensitivity differences between groups. Future studies would benefit from a more in depth comparison of multimodal conditions including an additional combination of old incongruent stimuli.
