# Supplementary material for: Enhanced voice recognition in musicians
Source: PLoS One. 2025 May 19;20(5):e0323604. doi: 10.1371/journal.pone.0323604 (PMC12088071; doi:10.1371/journal.pone.0323604)
Supplement: Attachment — Submitted filename: Response to Reviewers_R2.pdf [file pone.0323604.s003.pdf]

Enhanced voice recognition in musicians

Allison J. Sletcher, Stefania S. Moro, and Jennifer K. E. Steeves*

Response to Reviewers

Journal Requirements:

* **Data are published on York University's Dataverse. It can be found at the following DOI: https://doi.org/10.5683/SP3/L8OBJW**

Please review your reference list to ensure that it is complete and correct. If you have cited papers that have been retracted, please include the rationale for doing so in the manuscript text, or remove these references and replace them with relevant current references. Any changes to the reference list should be mentioned in the rebuttal letter that accompanies your revised manuscript. If you need to cite a retracted article, indicate the article's retracted status in the References list and also include a citation and full reference for the retraction notice.
Response: Reference list has been reviewed and verified.

Reviewer #1: This manuscript revision shows significant improvements. Specifically, I noticed better clarity in voice/person recognition terminology (cf. speech or linguistic processing), the methods have been updated with additional details, and a new analysis was added to evaluate possible differences in stimulus category sensitivity across the participant groups. I have a few more comments below that, if addressed, would further clarify aspects of this manuscript.

Major:
1. Lines 272-273 state: "There is no relationship between hours of weekly practice and sensitivity to voices for person identification." However, the stats in Table 1 and discussion suggest otherwise: e.g., lines 304-306: "this ability is related to the extent of experience as measured by hours of weekly practice and years of training"; lines 312-314: "better unimodal auditory sensitivity for voices is positively correlated with years of musical experience, as well as hours of practice". Please resolve these contradictions.
Response: Thank you for noticing this error. The results have been corrected to reflect the table and discussion.

2. Lines 294-295: "Non-musicians demonstrated a strong trend for enhanced auditory sensitivity for voices (M = 2.05, SD = 0.61, 95% CI [1.84, 2.26]) compared to object sounds", but Figure 3 and lines 322-323 state "On the other hand, non-musicians show a strong trend for better sensitivity to object sounds compared to voices." Please correct the first statement if the latter are indeed correct.
Response: You are correct that the latter statements are correct. The results have been corrected to reflect this.

Minor:
3. Line 73: Speech-in-noise cognition—is this intended to say "recognition"?
Response: Corrected.

4. I am a little confused by the wording "by replacing their perfect score with a constant" (line 191). This suggests to me that scores of 100% were replaced by a value of 0.025, but I am not certain that was the intended meaning.
Response: Yes, that is correct. Perfect scores were adjusted by either adding 0.025 (in the case where the participant got 0) or subtracting 0.025 (in the case where the participant got 100). We have attempted to clarify this by updating the text to the following: *Participants with perfect scores were adjusted based on recommendations by Macmillan and Creelman [60], by adjusting their score by a constant value (-0.025 in the case where participants scored 100).*

5. Line 198: "vsm" in parentheses—per the tracked changes version, I think this was meant to state "Musicians vs. Non-Musicians".
Response: Corrected.

6. The authors' response letter suggests Figure 1 was kept in the manuscript, but I did not see the original figures 1 & 2 in my copy, nor the corresponding object recognition figure. In the first manuscript version, I did find these helpful as a reader. It would be nice to add them back in if they can still be included per editor/publishing requirements.
Response: In this case there is a conflict with editor/publishing requirements. Due to copyright issues for the car stimuli images we have had to remove the figures from the paper. We have addressed your comment while still adhering to publishing requirements by re-inserting the schematic diagrams but with different car images from an open source dataset to be used for illustrative purposes.

Reviewer #2: The authors have satisfactorily addressed my previous comments. The article is a valuable contribution.
Response: Thank you!
